# How We Evaluate and Treat Leukemic Presentations of Mature T-Cell Lymphomas

**DOI:** 10.3390/cancers18060965

**Published:** 2026-03-17

**Authors:** Arjun Ravishankar, Vinisha Somaya, Haris Qureshi, Ahmad Kiwan, Francesca Montanari, Michael Girardi, Francine Foss, Tarsheen Sethi

**Affiliations:** 1 Department of Medicine, Division of Hematology-Oncology, Yale University School of Medicine, New Haven, CT 06510, USA; arjun.ravishankar@yale.edu (A.R.); haris.qureshi@yale.edu (H.Q.); ahmad.kiwan@yale.edu (A.K.); francesca.montanari@yale.edu (F.M.); francine.foss@yale.edu (F.F.); 2 Department of Surgery, University of Michigan Medical School, Ann Arbor, MI 48109, USA; vsomaya@umich.edu; 3 Department of Dermatology, Yale University School of Medicine, New Haven, CT 06510, USA; michael.girardi@yale.edu

**Keywords:** T-cell leukemia, T-cell lymphoma, T-cell prolymphocytic leukemia, adult T-cell leukemia/lymphoma, T-cell large granular lymphocytic leukemia, Sézary syndrome

## Abstract

T-cell non-Hodgkin lymphomas, which arise from post-thymic mature T cells, constitute approximately 10–15% of all non-Hodgkin lymphomas. Their leukemic presentations, referred to here as mature T-cell leukemias, are relatively uncommon and present significant diagnostic and therapeutic challenges requiring an informed approach to diagnosis and management. The initial presentation is often persistent T-cell lymphocytosis that must be distinguished from reactive (non-malignant) causes. Unlike B-cell lymphocytosis, where clonality usually indicates malignancy, T-cell clonality can be detected in benign conditions such as autoimmune disorders and viral infections. Thus, establishing clonality is helpful but not sufficient, and a systematic diagnostic approach integrating clinical features, morphology, immunophenotype, and molecular findings is critical. This review outlines our approach to the diagnosis and treatment of four major subtypes of mature T-cell leukemias: T-cell prolymphocytic leukemia (T-PLL), adult T-cell leukemia/lymphoma (ATLL), T-large granular lymphocytic leukemia (T-LGL), and Sézary syndrome (SS). Each section includes a discussion of clinical features, workup, and treatment options.

## Simple Summary

Mature T-cell lymphomas (TCLs) are classified based on their predominant site of involvement as peripheral TCLs, cutaneous TCLs, mature T-cell leukemias and extranodal TCLs. Mature T-cell leukemias are characterized by clonal T-cell lymphocytosis but the differential diagnosis of clonal T cells includes non-malignant conditions. Furthermore, the diagnostic evaluation of mature T-cell leukemias can be challenging and treatment is specific to subtypes, making it important to follow a structured approach to obtain an accurate diagnosis. In this review we outline a comprehensive approach to diagnosis and management of mature T-cell leukemias.

## 1. Diagnostic Approach to T-Cell Lymphocytosis

The initial challenge is determining if a T-cell lymphocytosis is reactive or malignant. Persistent elevation of absolute T-cell count with an abnormal immunophenotype on flow cytometry raises concern for a clonal T-cell proliferation. However, transient clonal or polyclonal expansions can occur in viral infections (e.g., EBV, CMV), chronic immune stimulation (autoimmune diseases), or after bone marrow/organ transplantation. The key distinguishing features are clinical context (e.g., HTLV-1 seropositivity points toward ATLL), the magnitude and persistence of lymphocytosis, the presence of associated cytopenias or organomegaly, and clonality assessment [1,2].

**Figure 1:**
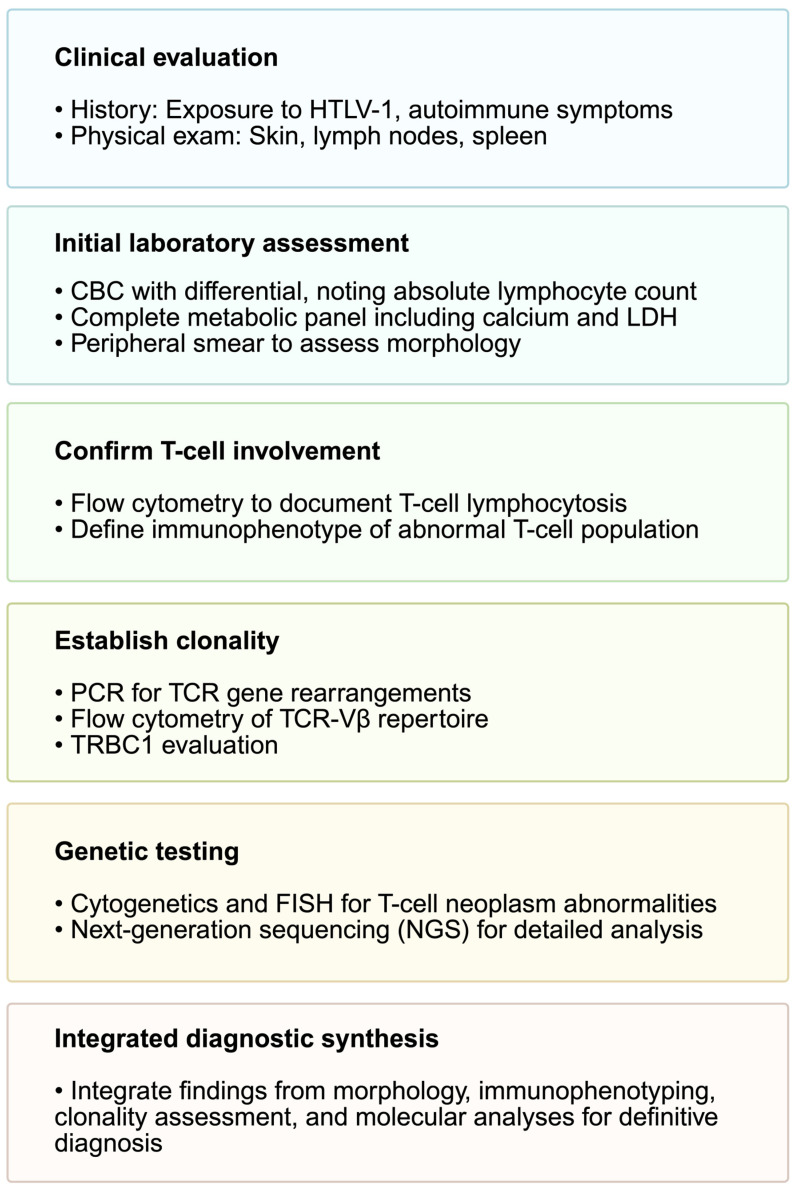
Initial diagnostic approach to T-cell lymphocytosis.

Non-malignant causes of polyclonal or monoclonal T-cell lymphocytosis include autoimmune diseases, infections, and inflammatory processes. These are typically polyclonal, transient, and associated with a clear precipitant. However, persistent immune stimulation can occasionally lead to the emergence of stable, oligoclonal or monoclonal T-cell populations. These so-called T-cell clones of unknown significance (T-CUS) often exhibit a T-LGL phenotype and need to be followed to monitor for progression to a T-cell malignancy [3,4]. Our diagnostic approach to T-cell lymphocytosis is summarized in Figure 1.

### 1.1. Initial Workup

All patients should have a thorough history (including exposure history for HTLV-1, symptoms of autoimmune disease, etc.) and physical exam (noting skin lesions, lymphadenopathy, hepatosplenomegaly). Labs include a complete blood count with differential, comprehensive metabolic panel (especially calcium levels for ATLL, as hypercalcemia is common) and LDH. Uric acid and tumor lysis labs should be obtained at baseline. Flow cytometry on peripheral blood is essential for immunophenotyping of the lymphocytes and clonality assessment to determine whether the abnormal T-cell population is monoclonal or polyclonal. Serologies for HTLV-1 (if ATLL is suspected) and other relevant viruses (HIV, EBV, hepatitis B and C, CMV, etc.) should be sent.

### 1.2. Staging and Tissue Biopsy

Bone marrow biopsy is generally indicated to assess morphology, pattern of involvement, and cytogenetics. Skin biopsies are required if cutaneous lesions are present, and in SS, staging includes assessment of total body skin involvement and lymph node biopsy if nodes are enlarged to rule out large-cell transformation. Imaging with contrast-enhanced CT or PET/CT is useful to evaluate the extent of nodal and extranodal disease, particularly in ATLL (to evaluate lymphadenopathy and organ infiltration) and T-PLL (which can have abdominal lymphadenopathy or serous effusions). Whole-body PET-CT is the preferred modality for staging CTCL/SS patients. We also recommend making an individualized decision to obtain whole-body PET-CT for patients with clonal T-cell lymphocytosis, since PTCL-NOS is in the differential. Red flags for malignancy may include clone size, atypical immunophenotype, presence of somatic mutations, presence of cytopenias, lymphadenopathy, splenomegaly and constitutional symptoms prompting additional work up in these patients.

### 1.3. Immunophenotype

Characteristic immunophenotypic and molecular features may aid in diagnosis. For example, T-PLL cells are typically CD4+ CD8± (often dual-positive) with strong CD7 and CD52 expression. ATLL cells are CD4+ CD25+ with frequent loss of CD7 and expression of CCR4. T-LGL leukemia involves CD3+ cytotoxic T cells, which are usually CD8+ CD57+. Sézary syndrome cells are atypical CD4+ T cells that co-express skin-homing markers (CCR4, CLA) and typically lose CD7 and CD26. Table 1 summarizes key immunophenotypic and genetic features of these entities.

### 1.4. Clonality Assessment

Clonal T-cell populations can be identified by T-cell receptor (TCR) gene rearrangement studies or flow cytometric Vβ repertoire analysis. Detection of a dominant TCR gene rearrangement confirms clonality, but as noted, not all T-cell clones are malignant. Flow cytometry for TCR-Vβ chain usage can also detect clonality; a restricted Vβ family indicates a clonal population. In practice, demonstration of clonality supports the diagnosis of a T-cell neoplasm when correlated clinical and pathologic findings are present [5,6]. More recently, TRBC1 expression by immunohistochemistry (IHC) or flow cytometry has been evaluated as a method to establish clonality [7,8]. TRBC2 evaluation by flow cytometry can also assist in T-cell clonality assessment but is not yet routinely used in clinical practice. The caveat of testing for TRBC-restricted T-cell subsets by flow cytometry is the detection of T-CUS in patients without T-cell neoplasms, which can lead to overtreatment [9].

### 1.5. Cytogenetics and Molecular Genetics

Specific genetic features carry diagnostic and prognostic significance. For T-PLL, FISH or karyotype help detect inv(14)(q11;q32) or t(14;14)(q11;q32), which juxtaposes the *TCL1* gene on chromosome 14 (14q32) with the T-cell receptor alpha/delta (*TRA/D*) locus on chromosome 14 (14q11.2), or t(X;14)(q28;q11) which causes the *MTCP1* gene on chromosome X to be overexpressed. In T-LGL, somatic mutations in *STAT3* are found in 30–50% of cases and help support the diagnosis. Comprehensive molecular profiling (targeted NGS panels) can provide additional diagnostic and prognostic information (e.g., TP53, ATM, *JAK1* or *JAK3* mutations in T-PLL, *RHOA* and other epigenetic mutations in *ATLL*, *STAT5B* in aggressive T-LGL) that can refine prognosis and guide therapy.

**Table 1:** Diagnostic features of T-cell leukemias.

Subtype	Clinical Features	Morphology	Immunophenotype (Key Markers)	Genetics/Molecular Findings	Differential Diagnosis
**T-Cell Prolymphocytic Leukemia (T-PLL)**	Rapidly progressive lymphocytosis (>30 × 10^9^/L), massive splenomegaly, lymphadenopathy; extranodal involvement (skin, serous effusions) is common.	Small-to-medium prolymphocytes; highly condensed chromatin, prominent nucleolus.	CD2+, CD3dim, CD5+, CD7+; Strong CD52+; CD4+/CD8− (65%); CD34−, CD25−, TdT−.	Inv(14)(q11q32) or t(14;14)(q11;q32) (TCL1 overexpression) (>90%); ATM deletion/mutation (80–90%); JAK3/STAT5B activating mutations.	T-cell acute lymphoblastic leukemia (T-ALL)-TdT+ and CD34+, CD1a+; Peripheral T-cell lymphoma, not otherwise specified (PTCL-NOS) in leukemic phase, typically CD52− and does not have chr14 abnormalities
**Adult T-Cell Leukemia/Lymphoma (ATLL)**	Aggressive (acute/lymphomatous) presents with hypercalcemia, organ infiltration; indolent (chronic/smoldering) presents with mild lymphocytosis, skin lesions.	“Flower cells” or “cloverleaf nuclei” (hyperlobated, abnormal lymphocytes).	CD4+, CD25+, CD7−, CD8−, CD26−, CCR4+.	Evidence of HTLV-1 infection is required; somatic mutations in RHOA, TET2, TP53; PD-L1 overexpression.	Sézary Syndrome (SS) and PTCL-NOS-Both are HTLV-1 negative, and CD25 dim/negative
**T-Large Granular Lymphocytic Leukemia (T-LGL)**	Chronic, indolent course (median OS 9–10 years); symptomatic cytopenias (severe neutropenia, transfusion-dependent anemia); association with autoimmune conditions (RA).	Large Granular Lymphocytes (LGLs); abundant cytoplasm, azurophilic granules; modest lymphocytosis (often >2.0 × 10^9^/L).	CD3+, CD8+, CD57+, CD16+ (Cytotoxic T-cells); CD28−; aberrant loss of CD5 or CD7.	Somatic mutations in STAT3 (30–70%), or STAT5B (less common and more aggressive); DNMT3A, TET2.	Reactive/polyclonal LGL expansions (e.g., secondary to CMV, EBV, HIV, or post-allogeneic transplant)—need to confirm clonality by flow or PCR; T-cell clones of uncertain significance (T-CUS)—lack symptomatic cytopenias or autoimmune syndromes; Chronic lymphoproliferative disorder of NK cells (CLPD-NK)—NK lineage cells are CD3−, TCR-, CD16+, CD56+
**Sézary Syndrome (SS)**	Aggressive leukemic CTCL; triad of diffuse erythroderma (>80% BSA), generalized lymphadenopathy, intense pruritus.	Atypical lymphocytes (Sézary cells) with highly grooved, convoluted “cerebriform” nuclei in blood.	CD3+, CD4+, CD7−, CD26−, CD8−; CD2−, CD5−; often co-express CCR4 and CLA.	Usually numerous copy number alterations (CNAs) and complex karyotype. Recurrent deletions: TP53, RB1, DNMT3A; mutations: TET2, MAPK1, TNFRSF1B.	Leukemic mycosis fungoides (MF)—typically evolves from pre-existing skin patches/plaques, in most cases B0 or B1 disease; erythrodermic inflammatory dermatoses (atopic dermatitis, drug eruptions)—lack monoclonal T-cell population with aberrant immunophenotype

## 2. T-Cell Prolymphocytic Leukemia (T-PLL)

### 2.1. Introduction

T-cell prolymphocytic leukemia (T-PLL) is a rare and aggressive mature T-cell leukemia first described in 1973 [10]. The age-adjusted incidence is 0.04 cases per 100,000 person-years in the US with a median age at diagnosis of 67 years and a slight male predominance [11].

The disease is characterized by activation of the T-cell leukemia/lymphoma 1 (*TCL1*) oncogene family on chromosome 14, typically through an inv(14)(q11.2q32) or t(14;14)(q11;q32), which juxtaposes the *TCL1* gene with the powerful T-cell receptor gene enhancers, leading to its overexpression. This is frequently accompanied by mutations or deletions of the *ATM* tumor suppressor gene on chromosome 11q, which occur in up to 90% of cases. Dysregulation of the JAK-STAT pathway, often via activating mutations in *JAK3* or *STAT5B*, is present in a majority of patients and contributes to the aggressive phenotype [12]. Abnormalities in the TCL-1 pathway can be detected by protein expression (TCL-1 by IHC or flow cytometry) or by genetic abnormalities in chromosome 14 by karyotype/FISH studies, as discussed below. TCL-1 family negative T-PLL has also been described and characterized by typical clinical presentation, morphology and molecular characteristics.

### 2.2. Clinical Features and Diagnosis

Patients typically present with rapidly progressive lymphocytosis, significant splenomegaly, and lymphadenopathy, although a few cases may be initially indolent with mild leukocytosis and slow clinical progression. As the disease becomes more advanced, extranodal involvement of the skin, bone marrow, and serous cavities (pleural or ascitic effusions) is common.

The diagnosis of T-PLL was standardized in 2019 by the T-PLL International Study group (TPLL-ISG) consensus criteria [13], which established a multiparametric set of criteria. These criteria are agreed upon by the fifth edition of the World Health Organization Classification of Haematolymphoid Tumours and include T lymphocytosis (>5 × 10^9^/L) with appropriate phenotype, T-cell monoclonality and the presence of genetic aberrations, including structural variants with breakpoints affecting the *TCL1A* or *MTCP1* locus or expression of TCL1 [14].

- **Morphology and Immunophenotype**: Peripheral blood shows a proliferation of small-to-medium-sized “prolymphocytes” with condensed chromatin and a visible nucleolus. T-PLL is distinguished by its immunophenotypic profile, which includes CD2+, CD5+, and CD7+ markers. It may also exhibit weak surface CD3 expression, while notably lacking CD25 and natural killer (NK) cell markers, an important distinction from other mature T-cell leukemias. Most T-PLL cases (65%) present with a CD4+/CD8− profile, whereas less common variants include CD4−/CD8+ (13%) and CD4+/CD8+ (21%). A defining characteristic of T-PLL is strong CD52 expression and the absence of CD34, TdT, and CD1a in its prolymphocytes, helping to differentiate it from T-acute lymphoblastic leukemia/lymphoblastic lymphoma (T-ALL/LBL).
- **Genetic testing**: T-PLL cells show clonal rearrangement in *TCR* genes (*TRB* or *TRG*). Cytogenetic studies identify complex karyotypes in about 80% of cases and characteristic recurrent genetic abnormalities involving the TCL-1 gene family. FISH commonly (>90%) detects characteristic abnormalities, such as inv(14) or t(14;14). Other chromosome abnormalities may be seen in chromosomes 11q, 8, 12p and 6. Molecular testing shows mutations in key genes, including *ATM* (11q23 deletion/missense mutation in 80–90% cases), *TP53* deletion or mutation, JAK3 (negative prognostic significance), and *STAT5B*, with JAK-STAT pathways being abnormal in up to 75% cases. Correct identification of T-PLL is essential, as its treatment strategies differ significantly from those for other aggressive T-cell neoplasms, where cyclophosphamide, doxorubicin, vincristine, and prednisone (CHOP)-like regimens are used, which are ineffective in T-PLL.

### 2.3. Treatment Overview and Our Approach to T-PLL

The TPLL-ISG consensus criteria for active T-PLL encompass disease-related constitutional symptoms, symptomatic bone marrow failure, rapid enlargement of lymph nodes/spleen, high lymphocyte count of >30 × 10^9^/L with rapid lymphocyte doubling time and extranodal involvement by T-PLL. In addition, the consideration of comorbidities using a scale like the Cumulative Illness Rating Scale-Geriatric (CIRS-G) is recommended. We consider HLA typing and candidacy of allogeneic transplant at the time of initial presentation. In fit patients who are candidates for transplant and meet criteria for active disease, we proceed with induction therapy and consolidate with allogeneic stem cell transplant (alloSCT). In select cases, such as young fit patients who do not yet meet criteria for active disease, we have a risk vs. benefit discussion of treatment without awaiting further progression due to several factors: progression of T-PLL is inevitable and with the lack of curative options without an allogeneic stem cell transplant, the disease has poor prognosis; disease activity can change quickly upon progression and the limited effective induction options may not result in a timely and/or deep response prior to alloSCT.

- **Frontline therapy:** There are limited prospective studies in T-PLL based on which the anti-CD52 monoclonal antibody alemtuzumab and fludarabine, mitoxantrone, cyclophosphamide (FMC), with or without alemtuzumab, are the most used induction regimens. Single-agent alemtuzumab is effective with an ORR of 90%, but responses are often short-lived with eventual relapses. The median PFS is 8–12 months with most patients relapsing within 2 years [15,16]. Adding alemtuzumab to FMC also resulted in an ORR of 92% with a median PFS of 11.9 months and median OS of 17.1 months; however, this combination is associated with greater toxicity [17]. Prophylaxis against Pneumocystis jirovecii pneumonia (PJP) and herpesvirus infections is a critical component of patient management. Frontline management approaches and outcomes have been summarized in Table 2.

**Figure 2:**
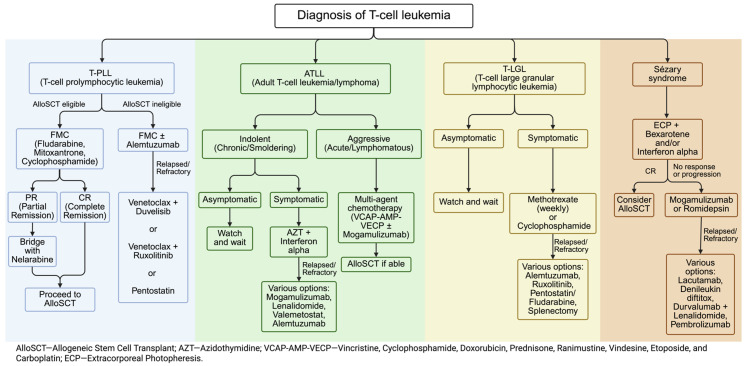
Approach to T-cell leukemia treatment.

The most commonly recommended first-line regimen for T-PLL, irrespective of alloSCT candidacy, is alemtuzumab, based on its high efficacy. However, in contrast with fludarabine, which follows predictable kinetics, alemtuzumab shows nonlinear elimination kinetics, wherein systemic clearance decreases with repeated administration due to decreased receptor-mediated clearance as CD52 receptors in the periphery are depleted. Small case series have demonstrated the potential impact of this in T-PLL patients undergoing alloSCT [18]. Due to this and the practical dilemma of losing response if there is a delay between the last alemtuzumab dose and alloSCT, our institutional practice is to use FMC for induction in these patients. In patients who are in <CR, we bridge with nelarabine based on early phase data [19]. Nelarabine is FDA-approved for T-ALL/LBL but not for T-PLL. Nelarabine can be associated with neurotoxicity and myelosuppression and should be used with caution at centers that are experienced in its use. Our approach is summarized in Figure 2.

- **Relapsed disease:** The prognosis of R/R T-PLL is poor with limited options, including retreatment with first-line options and pentostatin. The potential novel agents being considered include BCL-2 inhibitor Venetoclax, JAK inhibitors (esp. JAK3). Recent reports from a phase 1 study of combination ruxolitinib and duvelisib showed a 60% ORR in T-PLL patients and is being explored further [20].

**Table 2:** Frontline management and outcomes of T-cell leukemias.

Subtype	Disease Stage/Indication	Frontline Regimen/Strategy	Key Outcome Data (ORR/PFS/OS)	Additional Comments
**T-PLL**		FMC (Fludarabine, Mitoxantrone, Cyclophosphamide) followed by Alemtuzumab (Anti-CD52) consolidation. Alemtuzumab monotherapy if not fit enough for combination chemotherapy.	FMC + Alemtuzumab–ORR: 92%; median PFS: 11.9 months; median OS: 17.1 months [17]. Alemtuzumab monotherapy-ORR: 90%; median PFS: 8–12 months; high relapse risk within 2 years [16].	AlloSCT is the only established curative option. FMC preferred over Alemtuzumab prior to transplant due to risk of prolonged T-cell depletion. PJP and herpes prophylaxis mandatory.
**ATLL**	Indolent (chronic/smoldering), symptomatic	Azidothymidine (AZT) + Interferon-alpha (IFN-α)	ORR: 86%, CR 53% [21].	Watch-and-wait if asymptomatic
	Aggressive (acute/lymphomatous), fit patients	Intensive multi-agent chemotherapy such as LSG15 regimen (VCAP-AMP-VECP) ± Mogamulizumab (Anti-CCR4). Mogamulizumab + CHOP for elderly	LSG15 3-yr OS: 24% (vs. CHOP 13%) [22]; adding Moga increased CR rate from 33% to 52% [23]. Early AlloSCT assessment is critical (3-yr OS 34−39%) [24].	For CD30+ patients, BV-CHP is an alternative. CNS prophylaxis recommended for all patients.
**T-LGL**	Symptomatic cytopenias/autoimmunity	Low-dose weekly Cyclophosphamide or Methotrexate (MTX)	ORR: 38% [25] (higher in retrospective series). Responses can take 3–6 months.	Watch-and-wait if asymptomatic. STAT3 mutations correlate with MTX response.
	Refractory or Pure Red Cell Aplasia	Cyclophosphamide (oral daily) or Cyclosporine	ORR: 60–70%, CR:~30% [26]	Alternative first-line agent or second-line after MTX
**Sézary Syndrome (SS)**	Disseminated skin and blood disease (Stage IV)	ECP + IFN-α ± Bexarotene Romidepsin Mogamulizumab	Triple therapy with ECP, IFN, and retinoids is associated with longest time to next treatment (TTNT) [27]	Therapy aimed at disease control/QoL. AlloSCT reserved for selected patients (5-yr OS 30−50%).

## 3. Adult T-Cell Leukemia/Lymphoma (ATLL)

### 3.1. Introduction

Adult T-cell leukemia/lymphoma (ATLL) is a rare mature T-cell lymphoid malignancy that is etiologically linked to the Human T-cell lymphotropic virus type 1 (HTLV-1), an oncogenic virus endemic in Japan, the Caribbean, and parts of South America and Africa [28,29]. The disease typically manifests after decades of viral latency, with a small proportion of HTLV-1 carriers developing ATLL (estimated lifetime risk of approximately 1–5%). Higher proviral load of infected peripheral blood mononuclear cells (PBMCs) is associated with risk of malignant transformation. In the United States, 2148 ATLL cases were diagnosed from 2001 to 2015 [30].

### 3.2. Clinical Features and Diagnosis

ATLL can present as four clinical subtypes: acute, lymphomatous, chronic, and smoldering. The acute and lymphomatous subtypes are the most aggressive, characterized by high tumor burden, organ infiltration, hypercalcemia, and poor prognosis. The smoldering and chronic subtypes are more indolent and may present with skin lesions, mild lymphocytosis, and a more protracted clinical course. In the context of this review, the smoldering and acute forms present with T-cell lymphocytosis.

The diagnosis of ATLL is based on a combination of clinical presentation, morphology and immunophenotype of the cancer cells along with evidence of HTLV-1 infection.

- **Morphology and Immunophenotype:** Peripheral blood smears from patients with ATLL may reveal abnormal hyperlobated nuclei, commonly referred to as “cloverleaf” or “flower cells.” The malignant cells in ATLL are mature CD4+ T lymphocytes infected with HTLV-1. While the precise cellular origin remains controversial, ATLL cells characteristically exhibit a regulatory T-cell-like immunophenotype; typically expressing CD2, CD4, CD5 and CD25; have reduced CD3 expression; and are usually negative for CD7, CD8, and CD26. This immunosuppressive phenotype, including expression of immune checkpoint molecules such as PD-L1 in a subset of cases, contributes to immune evasion and the severe-immunocompromised-state characteristic of ATLL patients [31].
- **Genetic testing:** Genetic alterations can support the diagnosis but are not pathognomonic. Genetic analyses of ATLL cells have revealed somatic mutations in *RHOA* and *TET2*, as well as loss-of-function mutations in *TP53*. Additionally, overexpression of PD-L1 has also been reported [32].
- **HTLV-1 testing:** HTLV-1 testing is essential to distinguish ATLL from other TCLs, especially in endemic areas. HTLV-1 serology should be assessed by enzyme-linked immunoassay (ELISA) and confirmed by Western blot. PCR testing for HTLV-1 DNA can also be performed.

### 3.3. Treatment Overview and Our Approach to ATLL

The treatment of ATLL varies significantly based on the subtype of disease and clinical presentation, requiring tailored approaches.

- **Indolent ATLL (smoldering/chronic):** Asymptomatic patients can be managed with a “watch-and-wait” strategy, as conventional chemotherapy does not appear to improve their survival [21]. For symptomatic patients, a combination of interferon-alpha (IFN-α) and azidothymidine (AZT) has been the cornerstone of therapy. A meta-analysis of over 1000 ATLL patients treated with AZT/IFN reported an overall response rate of ~67% (CR ~33%), with patients with indolent subtypes having higher response than those with aggressive disease (ORR 86% vs. 68%, CR 53% vs. 25%) [33]. This regimen has also demonstrated efficacy in the acute leukemic subtype; however, it is notably ineffective in treating the lymphomatous subtype.
- **Aggressive ATLL (acute/lymphomatous):** Multi-agent chemotherapy, such as the EPOCH regimen (etoposide, prednisone, vincristine, cyclophosphamide, and doxorubicin), or more intensive protocols such as the Japanese LSG15 regimen: VCAP-AMP-VECP (vincristine, cyclophosphamide, doxorubicin, prednisone, ranimustine, vindesine, etoposide, and carboplatin) are commonly utilized. In a Japanese trial, biweekly CHOP achieved a complete remission (CR) rate of ~25%, whereas the intensive regimen achieved 40% CR. However, 1-year progression-free survival (28% with VCAP-AMP-VECP vs. 16% with CHOP) and 3-year OS (24% with VCAP-AMP-VECP vs. 13% with CHOP) were dismal [22]. Therefore, early evaluation for allogeneic stem cell transplantation (SCT) is a critical consideration, offering the potential for long-term remission in eligible patients. A retrospective study of nearly 600 patients who underwent alloSCT for ATLL showed 3-year OS of 39% for those who received myeloablative conditioning and 34% for reduced intensity conditioning, with a plateau of the OS plot indicating long-term survival [24]. A more recent retrospective study of EBMT registry data of 73 ATLL patients transplanted between 2004 and 2021 showed 2-year PFS and OS of 43% and 49% respectively [34] (Table 3).
  Mogamulizumab (Moga), a humanized anti-CCR4 monoclonal antibody, has shown significant activity in ATLL and is approved in Japan in both the frontline setting in combination with intensive chemotherapy and in the relapsed/refractory setting. In the frontline setting, adding Moga to the modified LSG15 regimen increased CR rates from 33% to 52%, albeit with similar PFS and OS and greater toxicity [23]. A recent trial adding Moga to CHOP for elderly (>66 years) and transplant-ineligible adults demonstrated 1-year PFS of 36% with CR and ORR of 64.6% and 91.7% respectively [35].
  For cases with CD30 expression, the BV-CHP regimen (brentuximab vedotin, cyclophosphamide, doxorubicin, and prednisone) provides a promising therapeutic option. Valemetostat is a novel EZH1/EZH2 inhibitor with significant activity in aggressive T-cell lymphomas and in ATLL. In a phase II study of relapsed/refractory ATLL patients conducted in Japan, the ORR was 48% with 20% CR [36]. Valemetostat remains a promising agent for ATLL [36].
- **Special considerations:** CNS prophylaxis is recommended in ATLL patients with high-risk features, as ATLL can involve the CNS. ATLL patients are highly immunosuppressed and prophylaxis for opportunistic infections such as PJP is essential. Immune checkpoint inhibitors have been associated with hyperprogression in ATLL and are contraindicated outside of clinical trials that can mitigate this effect.

Our treatment approach to ATLL stratifies this disease by subtype. For indolent subtypes (chronic/smoldering), we wait and watch until symptomatic, then treat with AZT and Interferon alpha. For aggressive subtypes (acute/lymphomatous), we treat with intensive chemotherapy and intrathecal methotrexate, consolidate with alloSCT when possible, and use Mogamulizumab in the relapsed/refractory setting. There are trials combining mogamulizumab with EPOCH (NCT05996185) and CNS prophylaxis, and another trial that combines belinostat with AZT/IFN [37] after frontline therapy as consolidation for patients with persistent circulating leukemia cells.

**Table 3:** Summary of data for allogeneic stem cell transplant in T-cell leukemias.

Parameter	ATLL [34]	T-PLL [38]	Sézary Syndrome [39]
**Overall Survival**	2-yr OS: 49%	4-yr OS: 30%	1-yr OS: 51%; 3-yr OS: 40%; 5-yr OS: 38%
**Progression-Free/Disease-Free Survival**	2-yr PFS: 43%	4-yr DFS: 26%	1-yr PFS: 42%; 3-yr PFS: 33%; 5-yr PFS: 26%
**Relapse Incidence**	49.8% at 2 years	42% at 4 years	47% (median time to relapse: 7.9 months)
**Non-Relapse Mortality**	7% at 2 years	32% at 4 years	18%
**Preferred Conditioning**	RIC (given older median age); MAC and RIC show similar outcomes	RIC without in vivo T-cell depletion (MAC associated with worse OS, HR 2.18)	RIC superior to MAC (OS 58% vs. 30%, p0.001)
**Key Prognostic Factors**	CR at transplant (HR 4.12 for OS if not in CR); haploidentical donors favorable	Age > 60 (HR 1.61); KPS < 90 (HR 1.53); disease status at transplant	CR at day +90 (relapse 20.8% vs. 70.6%); disease status at transplant
**Response to DLI**	Evidence of graft-versus-ATL effect	Can induce CR in relapsed patients	46% achieve CR after DLI for relapse
**Special Considerations**	Mogamulizumab before transplant increases severe GVHD risk; wait ≥50 days	Relapse therapy can be successful despite high relapse rates	Strong graft-versus-lymphoma effect; total skin electron beam may be incorporated

## 4. T-Cell Large Granular Lymphocytic (T-LGL) Leukemia

### 4.1. Introduction

T-LGL leukemia is a chronic, indolent lymphoproliferative disorder characterized by clonal expansion of cytotoxic T-cells (large granular lymphocytes). The molecular pathogenesis of T-LGL is multifactorial but, in many cases, is associated with constitutive activation of the JAK-STAT survival pathway, conferring a pro-survival, anti-apoptotic advantage to the clonal LGL population. The pathogenesis of neutropenia and anemia in T- LGL is multifactorial including both peripheral (antibody-mediated destruction and splenic sequestration) and central mechanisms (immune-mediated bone marrow suppression, e.g., FAS expression by leukemic cytotoxic T lymphocytes mediates direct cytotoxicity of hematopoietic precursors; in addition, T-cell-derived IFN-γ activates additional immune cells that contribute to the cytokine milieu) [40]. T-LGL has an incidence of 0.2 cases per 1,000,000 individuals (0.02 per 100,000) [41].

### 4.2. Clinical Features and Diagnosis

Many patients with T-LGL are asymptomatic and diagnosed incidentally, and the disease tends to follow a chronic course over many years. Symptomatic patients typically present with lymphocytosis or cytopenias, most commonly neutropenia (leading to recurrent infections) and anemia. T-LGL is strongly associated with autoimmune conditions, especially rheumatoid arthritis; up to 30% of patients have concurrent RA and neutropenia (Felty’s syndrome). Splenomegaly is seen in about half of cases, but lymphadenopathy is rare. Some cases may be subclinical and never require therapy, whereas others can cause severe neutropenia or transfusion-dependent anemia.

The diagnostic challenge is not merely to identify a clonal LGL population that can exist as an indolent clone of undetermined significance (T-CUS), but to link it to a resulting clinical syndrome that necessitates therapy.

- **Morphology and Immunophenotype:** Peripheral blood typically shows an increase in large granular lymphocytes with abundant cytoplasm and azurophilic granules. The absolute lymphocyte count may be modestly increased or normal, and the absolute LGL count is usually >2.0 × 10^9^/L but is not required for the diagnosis. Immunophenotyping reveals CD3+ CD8+ cytotoxic T cells that are usually CD57+ and CD16+ and CD4−, CD56−. A CD4+ CD8+/- CD56+ variant is often associated with *STAT5B* mutation. T-LGL cells characteristically lack CD28 and often have aberrant loss of either CD5 or CD7. Clonality is confirmed by TCR gene rearrangement studies or flow cytometric Vβ analysis.
- **Genetic testing:** Somatic mutations in the *STAT3* gene are present in 30–70% of cases and can support the diagnosis. *STAT3* mutations (especially Y640F) also correlate with responsiveness to methotrexate therapy. Less commonly, *STAT5B* mutations are found and may portend more aggressive disease. Mutations are also seen in epigenetic modifiers such as *DNMT3A*, *KMT2D* and *TET2* [42].

### 4.3. Treatment Overview and Our Approach to T-LGL Leukemia

All patients with T-CUS and many T-LGL patients do not require immediate treatment. Observation is appropriate for asymptomatic patients with mild cytopenias. Indications for therapy include severe neutropenia (ANC < 0.5 × 10^9^/L or recurrent infections), symptomatic anemia or transfusion dependence, or disabling autoimmune manifestations. First-line treatment is low-dose immunosuppression rather than aggressive chemotherapy, as T-LGL behaves more like a chronic immune-mediated disorder. The most widely used first-line agents are:

- **Methotrexate (MTX):** Low-dose oral methotrexate (10 mg/m^2^ weekly) is a standard initial therapy. In the prospective ECOG E5998 trial, MTX yielded an ORR of 38% [25], but other case series report response rates of ~50%. Responses with MTX can take 3–6 months to manifest. MTX can improve neutropenia and anemia, and helps arthritis in cases with RA. It is generally well tolerated; side effects include liver enzyme elevations and mucositis.
- **Cyclophosphamide:** Oral cyclophosphamide (50–100 mg daily for 6–12 months) is another first-line option, particularly in patients with pure red cell aplasia or those failing MTX. Cyclophosphamide has reported ORR ~60–70%, with CR rates ~30% [26]. Like MTX, responses can be slow, and treatment is often continued for 6+ months if tolerated.
- **Cyclosporine (CsA):** Cyclosporine can be effective, with ORR ~40–60% in various reports. It is used less often as a first-line agent due to its side effects (renal impairment, hypertension) but is an option, especially if anemia is prominent or in patients with concurrent rheumatoid arthritis refractory to MTX. Responses correlate with HLA-DR4 positivity in some studies.

A recent randomized trial compared MTX to cyclophosphamide and showed a better response after 4 months for cyclophosphamide vs. methotrexate (24% vs. 12% CR and 73% vs. 32% ORR) without any difference in toxicity [43]. If first-line therapy fails or loses effect, patients are switched to an alternate single agent. Combination immunosuppression is generally avoided upfront due to infection risk but can be considered in refractory cases. Granulocyte colony-stimulating factor (G-CSF) support is given as needed for severe neutropenia alongside definitive therapy. Our approach to T-LGL is to observe if it is asymptomatic and use low-dose MTX or cyclophosphamide as needed for patients who manifest with clinically significant cytopenias. For refractory cases, options include Alemtuzumab (which has shown responses even in heavily pre-treated patients), JAK2 inhibitor ruxolitinib, purine nucleoside analogs (such as pentostatin or fludarabine, very limited data) or, in rare cases, splenectomy (in transfusion-dependent anemia or refractory neutropenia).

## 5. Sézary Syndrome

### 5.1. Introduction

Sézary syndrome (SS) is an aggressive leukemic variant of cutaneous T-cell lymphoma (CTCL), defined by a triad of erythroderma (generalized red scaly skin), generalized lymphadenopathy, and clonal T cells in skin, lymph nodes, and peripheral blood. SS is associated with Th2 cytokines such as IL-4 and IL-5, as well as increased production of IL-10 and TGF-beta, which contributes to impaired cell-mediated immunity, eosinophilia, and atopy-like symptoms [44]. The cytokines promote malignant cell survival and proliferation, create a proinflammatory microenvironment, enhance skin tropism through chemokine receptor signaling (CCR4/CCL17, CCR10/CCL27), and suppress anti-tumor immunity. Sézary syndrome usually occurs in older adults (median age ~60) and is considered stage IV CTCL (Erythrodermic CTCL with B2 blood involvement).

### 5.2. Clinical Features and Diagnosis

SS typically presents with diffuse erythroderma accompanied by intense pruritus and often palmoplantar keratoderma and alopecia. Patients often have dramatic skin symptoms and suffer infections due to skin barrier breakdown and immunodeficiency, leading to increased potential for bacteremia. They are also at higher risk for second malignancies, especially other lymphomas and cutaneous malignancies [45].

A skin biopsy of erythrodermic skin typically shows a diffuse epidermal T-cell infiltrate (though sometimes indistinguishable from benign eczema or drug rash) and should be tested for T-cell clonality. The peripheral blood is characterized by atypical Sézary lymphocytes with cerebriform nuclei, which demonstrate an abnormal phenotype. Sezary cells are CD4+ T cells that typically lack expression of T-cell antigens such as CD7 and/or CD26 and often express CCR4, cutaneous lymphocyte antigen (CLA), and other skin-homing antigens. An absolute Sézary cell count of ≥1000/µL or a CD4:CD8 ratio >10 with evidence of clonality is generally diagnostic of SS. TCR clonality must be tested, and the presence of a dominant circulating T-cell clone in blood that matches the skin clone confirms SS.

The consensus criteria for diagnosis of SS require a skin biopsy that is diagnostic, erythroderma covering > 80% of body surface area, and >1000/μL of Sézary cells or CD4+ CD26− or CD4+ CD7− lymphocytes [46].

### 5.3. Distinguishing Sézary Syndrome from Leukemic Mycosis Fungoides

While both SS and erythrodermic mycosis fungoides (MF) can present with erythroderma and blood involvement, the critical distinguishing feature is the degree of blood involvement: SS is defined by B2 blood involvement (≥1000/μL Sézary cells), whereas erythrodermic MF typically has minimal or low-level blood involvement (B0: <250/μL Sézary cells). B1 (>250 to <1000/μL Sézary cells) or B2 disease can occur in MF patients, especially with more extensive skin involvement, and carries prognostic significance for survival and disease progression [47]. Blood tumor burden should be understood as a dynamic marker reflecting ongoing trafficking of malignant cells rather than a static staging parameter, and serial monitoring of blood tumor burden by flow cytometry can provide valuable information for assessing disease progression and treatment response [48].

### 5.4. Treatment Overview and Our Approach to Sézary Syndrome

The treatment of SS is challenging and typically not curative for most. Treatment involves multimodal care and aims to achieve disease control and improve quality of life. Key modalities include:

**Extracorporeal photopheresis (ECP):** ECP involves extracorporeal UVA irradiation of leukapheresed circulating mononuclear cells in the presence of a DNA-damaging agent, methoxy psoralen, followed by reinfusion of the treated mononuclear cells. The response rate to ECP is reported to be ~40–60% in Sezary patients [49]. ECP is often combined with systemic therapies like interferon or retinoids, which can enhance the efficacy of the treatment [50].

**Interferon-alpha (IFN-α):** IFN can induce disease regression in SS and is often given SC three times weekly. ORR of ~45% has been reported for IFN monotherapy in SS [51], and higher in combination (e.g., with photopheresis) [52]. IFN helps restore immune function and can lead to reduced tumor burden in the blood and skin. Responses have also been seen with IFN gamma.

**Bexarotene:** This oral retinoid (RXR agonist) is FDA-approved for CTCL. It produces partial responses in ~50% of advanced CTCL patients [53]. In SS, bexarotene is associated with clinical responses in blood and skin lesions. Side effects (hypertriglyceridemia, central hypothyroidism) require ongoing monitoring and administration of levothyroxine and lipid-lowering agents.

**Mogamulizumab:** Anti-CCR4 antibody approved for relapsed CTCL (MF/SS). A phase III randomized clinical trial of mogamulizumab vs. oral vorinostat was conducted in patients with relapsed/refractory MF/SS (MAVORIC trial) and reported an ORR of 37% (vs 2% with vorinostat), with 54% of SS patients achieving clearance of circulating Sézary cells [54]. Median PFS was ~7.7 months in SS, and adverse events included infusion reactions and immune-mediated skin rash, which can be confused with transient disease flares.

**Histone deacetylase (HDAC) inhibitors:** Romidepsin and vorinostat are FDA-approved for relapsed CTCL and have activity in SS. Romidepsin (IV, cyclic depsipeptide) achieved ORR ~34% (CR ~6%) in CTCL [55]. Improvement was reported in skin lesions and pruritus in SS, patients though effects on blood tumor burden were variable. Vorinostat (oral) has ORR ~30% in CTCL [56].

**Chemotherapy:** Single-agent or combination chemotherapy (e.g., gemcitabine, liposomal doxorubicin, chlorambucil/prednisone) can palliate disease but is typically short-lived in effect. We reserve systemic chemotherapy for rapidly progressive or tumor-stage disease not responding to other measures, or as a bridge to transplant.

**Brentuximab vedotin (BV):** BV is an anti-CD 30 antibody conjugated with the tubulin inhibitor monomethylauristatin E and can be used for those with CD30+ disease. In a phase II trial, BV showed an ORR of 70% with responses in the skin, blood, and lymph nodes [57]. The main toxicity of BV was peripheral neuropathy.

**Allo-HSCT:** Allogeneic transplant can be considered in SS patients who are fit to undergo the transplant procedure. While data is limited, patients can achieve prolonged remission post-transplant (5-year survival ~30–50% in selected series). Data is summarized in Table 3.

Our approach for SS is to start with ECP + IFN and/or bexarotene as first-line for disseminated skin and blood disease, since registry data shows that combination therapy is associated with the longest time to next treatment (TTNT) [27]. If there is inadequate response after ~6 months or progressive disease, we escalate to mogamulizumab or romidepsin. In a fit patient with responsive disease, we consider allo-HSCT consolidation. For refractory cases, newer experimental therapies may be tried (Table 4). One promising agent is lacutamab (IPH4102), an anti-KIR3DL2 antibody targeting a molecule highly expressed on Sézary cells. Phase 1 data showed a ~36% ORR in SS [58]. Another emerging therapy that was recently FDA-approved is Denileukin diftitox (“ONTAK”), an IL-2 diphtheria-toxin fusion protein that targets CD25; a recent trial reported a ~36% ORR in CTCL [59]. These novel therapies and others (e.g., checkpoint blockade combined with other immunotherapies to avoid tumor flare) are being investigated.

Despite these advances, Sézary syndrome remains incurable in most cases. Patients require continuous therapy and supportive care (skin emollients, antibiotics for infections, etc.). The median survival for stage IV SS is ~3 years, though long-term survivors exist. Combination and sequential therapies are used to maintain disease control. The importance of clinical trial participation in SS cannot be overstated, as new agents (e.g., anti-KIR3DL2, immune modulators) offer hope for improved outcomes.

**Table 4:** Novel agents/combinations and future directions.

Subtype	Novel Agent/Combination	Mechanism	Outcome Data/Status
T-PLL	**Ruxolitinib + Venetoclax**	JAK1/2 + BCL-2 inhibitor	ORR 73% with improved PFS in JAK-mutated group (5.6 mo. vs. 1.8 mo.) [60].
	**Ruxolitinib + Duvelisib**	JAK1/2 + PI3K-δ/γ inhibitor	ORR 60% in T-PLL patients [20].
ATLL	**Valemetostat**	EZH1/EZH2 inhibitor	ORR 48%, CR 20%, mDOR not reached [36]; approved in Japan for R/R ATLL
	**Lenalidomide**	Immunomodulatory, binds to cereblon (ubiquitin E3 ligase)	ORR 42%, CR 15%, mPFS 3.8 months, mOS 20.3 months [61]
T-LGL	**Ruxolitinib**	JAK1/2 inhibitor	ORR 86%, CR 14%, mDOR 4 months [62]
	**Alemtuzumab**	Anti-CD52 antibody	ORR 56%, CR 20% [63]
SS	**Lacutamab**	Anti-KIR3DL2 antibody, a molecule highly expressed on Sézary cells	ORR 37.5%, mPFS 8 months [58]
	**Denileukin diftitox**	Recombinant protein fusing the part of IL-2 that binds to the IL-2 receptor (IL-2R) with diphtheria toxin	ORR 36%, CR 9%, mDOR 8.9 months [59]
	**Durvalumab + Lenalidomide**	Anti-PD-L1 antibody + Immunomodulatory agent	ORR 75%, 73% PFS at 12 months [64]
	**Pembrolizumab**	Anti-PD-1 antibody	ORR 38%, CR 8% [65]

## 6. Conclusions

Leukemic presentations of T-cell lymphomas represent a heterogeneous group of rare disorders that pose significant diagnostic and therapeutic challenges. An accurate diagnosis requires careful correlation of clinical features, viral serologies in some cases, immunophenotyping, and clonality studies. Distinguishing malignant clonal proliferations from benign reactive expansions is a critical first step, requiring expert hematopathology input and often advanced molecular assays. Once the subtype is identified, management must be individualized: T-PLL demands urgent therapy and consideration of transplant in eligible patients, whereas T-LGL can often be managed conservatively with low-dose immunosuppression or even observation. ATLL treatment hinges on correctly classifying subtype and utilizing antiviral therapy for indolent disease versus multi-agent chemotherapy (and stem cell transplant) for aggressive disease. Sézary syndrome requires a multimodal approach combining skin-directed and systemic therapies to achieve disease control and maintain quality of life, as well as consideration of potentially curative allogeneic stem cell transplantation in patients with high-risk disease.

Despite the disparate strategies, a unifying principle is that conventional therapies have limited success in these diseases, and relapses are common. Allogeneic stem cell transplant offers a chance of long-term remission in T-PLL, ATLL, and SS, but is only feasible in patients who have a donor and are candidates for transplant. Newer targeted agents like mogamulizumab and valemetostat have shown significant activity in relapsed SS and ATLL respectively. Ongoing trials with BCL-2 inhibitors, JAK inhibitors, immune modulators, and monoclonal antibodies (Table 2 and Table 4) are beginning to yield encouraging results. Combining these novel agents with existing backbones (or with each other) may further enhance efficacy.

In the management of these patients, it is crucial to balance treatment intensity with patient fitness. Many patients are older adults with comorbidities, so gentler approaches (like oral agents in T-LGL or photopheresis in SS) are used when possible, reserving intensive therapies for those who can tolerate them. Supportive care, such as prophylaxis against infections, management of hypercalcemia in ATLL, skin care in SS, and growth factor support in T-LGL, remains an integral part of the overall treatment plan.

Lastly, given the rarity of these disorders, referral to specialized centers and enrollment in clinical trials is highly recommended. Multi-center collaborations (such as the T-PLL International Study Group, the United States Cutaneous Lymphoma Consortium and the International Society for Cutaneous Lymphoma, and the international ATLL consortiums) are helping define standard criteria and novel endpoints, which will ultimately facilitate the development of new therapies. The hope is that emerging targeted treatments and immunotherapies will gradually transform these historically refractory leukemic T-cell neoplasms into more manageable conditions, improving survival and quality of life for patients. In the meantime, an individualized, expert-driven approach as outlined in this review offers the best outcomes, integrating current evidence with clinical judgment for each unique patient scenario.
